# Structural, Functional, and Evolutionary Characterization of Major Drought Transcription Factors Families in Maize

**DOI:** 10.3389/fchem.2018.00177

**Published:** 2018-05-23

**Authors:** Shikha Mittal, Pooja Banduni, Mallana G. Mallikarjuna, Atmakuri R. Rao, Prashant A. Jain, Prasanta K. Dash, Nepolean Thirunavukkarasu

**Affiliations:** ^1^Division of Genetics, ICAR-Indian Agricultural Research Institute, New Delhi, India; ^2^Centre for Agricultural Bioinformatics, ICAR-Indian Agricultural Statistics Research Institute, New Delhi, India; ^3^Department of Computational Biology & Bioinformatics, J.I.B.B., Sam Higginbottom University of Agriculture, Technology and Sciences, Allahabad, India; ^4^National Research Centre on Plant Biotechnology, New Delhi, India

**Keywords:** drought, gene expression, gene interaction, maize, transcription factors

## Abstract

Drought is one of the major threats to the maize yield especially in subtropical production systems. Understanding the genes and regulatory mechanisms of drought tolerance is important to sustain the yield. Transcription factors (TFs) play a major role in gene regulation under drought stress. In the present study, a set of 15 major TF families comprising 1,436 genes was structurally and functionally characterized. The functional annotation indicated that the genes were involved in ABA signaling, ROS scavenging, photosynthesis, stomatal regulation, and sucrose metabolism. Duplication was identified as the primary force in divergence and expansion of TF families. Phylogenetic relationship was developed for individual TF and combined TF families. Phylogenetic analysis clustered the genes into specific and mixed groups. Gene structure analysis revealed that more number of genes were intron-rich as compared to intron-less. Drought-responsive *cis*-regulatory elements such as ABREA, ABREB, DRE1, and DRECRTCOREAT have been identified. Expression and interaction analyses identified leaf-specific bZIP TF, *GRMZM2G140355*, as a potential contributor toward drought tolerance in maize. Protein-protein interaction network of 269 drought-responsive genes belonging to different TFs has been provided. The information generated on structural and functional characteristics, expression, and interaction of the drought-related TF families will be useful to decipher the drought tolerance mechanisms and to breed drought-tolerant genotypes in maize.

## Introduction

Maize serves as a model system for genetic studies from simple inheritance, physical linkage, cytological crossing over, and recombination analyses to modern epigenetic silencing, genome imprinting, and transposition (Bennetzen and Hake, 2009). Although maize production is considerably affected by many biotic and abiotic stresses, drought is the major one (Leach et al., 2011). Being a water-intensive field crop, shortage of water severely limits the productivity of maize. The stress manifests through several symptoms, including increased leaf senescence and smaller leaves. Cellular damage and impaired carbon fixation during drought lead to imbalanced carbon assimilation and yield reduction (Hall et al., 1981). Drought is thus a serious threat to food security in all maize production systems.

During drought, plant TFs promote multiple responses at the molecular level by regulating gene expression and develop interactions with basal TFs at target gene promoters in response to developmental and intracellular signals, and control the target gene function either by activation or repression (Lu et al., 2012). Through drought-TF interfaces, the plants can tightly control gene expression in response to drought or water stress signals. Many drought-responsive TFs, including AP2, bZIP, NAC, HD-Zip, MYB, and NF-YA, play an active role in drought regulation, and the function of these genes is important for drought tolerance (Mittal et al., 2017; Thirunavukkarasu et al., 2017).

The structural and functional characterization of transcription factors have been studied in different crops. Physical mapping of WRKY in *Setaria italica*, NAC in *Brachypodium distachyon*, and MYB in *Arabidopsis* and rice revealed an uneven distribution of genes due to distinct duplication patterns (Katiyar et al., 2012; Muthamilarasan et al., 2015). Grouping of TFs into different families could be done based on 3D structural similarity in DNA-binding and acidic domains (Yilmaz et al., 2009). Phylogenetic studies on HD-Zip in maize, and bZIP in cucumber and rice also showed the formation of different clades on the basis of similarities and divergences (Baloglu et al., 2014; Mao et al., 2016). TF families have specific roles in plant response to abiotic stress; for example, the basic leucine zipper (bZIP) family consists of a common bZIP domain for DNA binding at the N terminus and a leucine-rich motif for dimerization at the C terminus and functions in an ABA-dependent manner by associating with ABRE (ABA-responsive *cis*-regulatory element) in its promoter regions (Zou et al., 2008). WRKY family has a conserved WRKY domain that binds to the W-box (a conserved stretch of 60 amino acids) to control the expression of genes (Rushton et al., 2010). All WRKY genes contain a conserved amino acid sequence (WRKYGQK) at the N terminus and a zinc finger motif at the C terminus. Several studies have shown that the role of WRKY in drought response is through modulation of an ABA-dependent pathway.

In maize, different TF families have been identified as responsible for modulating the gene regulation in response to drought stress. TFs such as MYB, NAC, WRKY, bZIP, bHLH, HD-zip *via* ABA-dependent or ABA-independent pathways play a significant role in drought tolerance. *ZmNF-YB2* in maize is shown to have an equal role as *AtNF-YB1* in *Arabidopsis* to confer improved crop performance under drought conditions (Nelson et al., 2007). bZIP plays a vital role in ABA signaling along with other functions in plant growth and abiotic stresses. *ZmbZIP72*, a bZIP TF in maize was over-expressed in various organs in response to drought, salinity, and ABA stress at seedling stage. AP2, ERF, dehydration-responsive element-binding protein (*DREB*), Cys2His2 Zinc Finger (C_2_H_2_ ZF), MYB, bHLH were identified as important TFs for drought tolerance (Ying et al., 2012). A set of 10 drought-responsive TF families (AP2, ERF, MYB, bZIP, bHLH, GRAS, WRKY, NAC, NF-YA, and NF-YB) have been identified in maize to regulate different physiological and molecular functions namely, stomatal regulation, hormone signaling, root development, and osmoregulation (Mittal et al., 2017).

The role of TFs in various stress tolerances in different crop plants including maize is well-documented. However, there is no substantial report on comprehensive structural and functional analysis of the major drought TF families in maize. Therefore, the present investigation has been designed to identify and characterize major TF families related to drought tolerance. These families were classified based on their putative DNA binding domain and the transcription regulatory domain (TRD) or transcriptional activation domain (TAD). The study covered the following aspects: detailed analysis of TF-specific domains, physical mapping of the TF families, prediction of gene functions, phylogeny, duplication events, paralogs, gene structure, *cis*-regulatory elements (CREs) and motifs analysis, expression assay, and protein-protein interaction analysis. A hypothesis on how different TFs by binding to *cis*-regulatory elements regulate drought tolerance in maize has been developed. The results provide insights into the role of TF families in relation to drought. Further a functional analysis has identified important TF genes which can be used as candidate for drought breeding.

## Materials and methods

### Identification of TF gene families and domain analysis in maize

A set of 15 major drought-related TF families namely, AP2, ARF, bHLH, bZIP, C2H2, ERF, GATA, GRAS, HD-Zip, HSF, MYB, NAC, NF-YA, NF-YB, and WRKY were selected for structural and functional characterization. These TF families were considered as major drought-related TF families (Thirunavukkarasu et al., 2014; Mittal et al., 2017; Van Gioi et al., 2017) and a total of 1,436 genes representing those families were selected from the Plant Transcription Factor Database 3.0 (http://planttfdb.cbi.pku.edu.cn) and in-house data (Thirunavukkarasu et al., 2014; Mittal et al., 2017). We noticed that although some member proteins lacked a specific TF family domain, they were nevertheless annotated as integral members of that TF family. Therefore, we excluded such redundant member proteins from consideration. To identify the number of domains present in a TF family protein, we searched the Pfam database (http://pfam.xfam.org) with an expectation cut-off (*E*-value) of 1.0, and only the significant domains found in the maize protein sequences were considered as valid domains.

### Chromosomal distribution

The chromosomal position of each gene encoding a maize protein was determined by Phytozome database (https://phytozome.jgi.doe.gov/pz/portal.html). Each TF family was then mapped onto the maize genome (*n* = 10). Genes were plotted separately onto 10 chromosomes of maize by using R package “*synbreed*.”

### Gene ontology

Nucleotide and protein sequences of the 1,436 TF genes and primary information about them, including the chromosome on which they are located, were retrieved from the Ensembl plant's website (http://asia.ensembl.org/index.html). GO annotations of all maize TF family proteins were executed using the Blast2GO server (https://www.blast2go.com). The Blast2GO annotation links the genes with the GO terms using hierarchical vocabularies. The GO terms have been classified into three categories: biological process, cellular components, and molecular function. The visual display of GO annotation has been drawn using WEGO tool.

### Phylogenetic analysis

Phylogenetic analysis was performed using full-length protein sequences to understand the evolutionary relationship among and within the TFs. To generate the phylogenetic tree for TFs families, amino acid sequences were imported into MEGA 6.06 to perform multiple sequence alignment with gap open and gap extension penalties of 10 and 0.1, respectively. Then the alignment file obtained using MSA was used for the construction of unrooted phylogenetic tree using neighbor-joining method through MEGA ver. 6.06 (http://www.megasoftware.net) and was rendered using iTOL (http://itol.embl.de).The reliability of the constructed unrooted tree was established using 1,000 rapid bootstrap replicates.

### Gene duplication and paralog identification

Information on gene duplication events, such as tandem duplication and block duplication, was retrieved using PLAZA, a tool (http://bioinformatics.psb.ugent.be/plaza/) available at Plant Genome Duplication Database (PGDD) (http://chibba.agtec.uga.edu/duplication). Each gene was aligned against the 1,436 genes, and paralogs with a minimum of 75% identity were searched using BLAST (https://blast.ncbi.nlm.nih.gov). Adjacent genes of the same sub-family located within 10 predicted genes apart or within 30 Kbp of each other were characterized as tandem duplications (Shiu and Bleecker, 2003; Du et al., 2013).

### Gene structure analysis

Gene structure or the exon-intron structure of the genes was identified using the Gene Structure Display Server 2.0 (http://gsds.cbi.pku.edu.cn). The genomic sequences were aligned with their corresponding coding sequences for identifying the exon-intron structure in GSDS 2.0. The genomic and coding sequences of the genes were extracted from Ensembl genomes BioMart (http://ensemblgenomes.org).

### Motif prediction

The conserved protein and nucleotide motifs for all 15 TF families were discovered using MEME 4.10.2, the multiple maximizations for motif elicitation analysis tool (http://meme-suite.org/doc/download.html). The MEME suite was used to search 5 motifs of each TF family with a base width of a minimum of 12 and maximum of 60 Kb and an *E*-value smaller than 0.01.

### Identification of *cis*-regulatory elements

Upstream promoter sequences (1 Kb) of all the major drought-related TF families have been retrieved using Phytozome Biomart (https://phytozome.jgi.doe.gov/pz/portal.html). These sequences were then used to predict and locate the *cis*-elements using PlantPAN (http://plantpan2.itps.ncku.edu.tw).

### Expression analysis of drought-responsive TF genes in *Zea mays*

Expression analysis of TF genes was performed using maize RNA-Seq data generated under drought stress. A total of two independent RNA-Seq experiments (Liu et al., 2015; Thatcher et al., 2016) available in the NCBI's SRA database for *Zea mays* were considered for *in-silico* expression analysis (Accession numbers: SRP061631 and SRP062027; https://www.ncbi.nlm.nih.gov). In the first experiment, a drought-tolerant maize genotype ZD619 was used to generate RNA-Seq data under drought stress and controlled condition using root, stem, and leaves. In the second experiment, RNA-Seq data was generated using B73 under drought stress in different growth stages such as V12, V14, V18, and R1. Expression data from the above-mentioned experiments were considered for the validation of genes.

### Protein interaction network analysis of drought-responsive TFs

Based on the expression data TF interactions were studied for the selected 269 drought-responsive genes. The STRING 10.0 (Search Tool for the Retrieval of Interacting Genes/Proteins) was employed to analyze and visualize the TF interaction network (http://string-db.org). The network was constructed using the confidence score of 0.5 to assure a high reliability of the interaction network and all other parameters were the default.

## Results and discussion

In the present study, major drought-related TF families have been selected on the basis of their role in drought tolerance in maize and in different species revealed by literature and in-house experiments (Thirunavukkarasu et al., 2014; Mittal et al., 2017; Van Gioi et al., 2017). For example, *SNAC1* gene confers drought tolerance in rice by regulation of downstream genes (You et al., 2014). NAC has been characterized in different species including Chinese cabbage, soybean, maize, rice, *Arabidopsis* and grape (Shao et al., 2015). The over-expression of *ANACO19, ANACO55*, and *ANACO72* elevated the drought expression in *Arabidopsis* (Joshi et al., 2016). In *Arabidopsis*, rice, and soybean, *AREB1* over-expression improves the drought tolerance (Oh et al., 2005; Barbosa et al., 2013; Yoshida et al., 2015). These major 15 drought-related TF families comprise of 1,436 genes of which, MYB family had the highest number of genes (281), followed by ERF (181), whereas NF-YA had the lowest (16) (Supplementary Table S1).

### Chromosomal distribution

**Figure 1:**
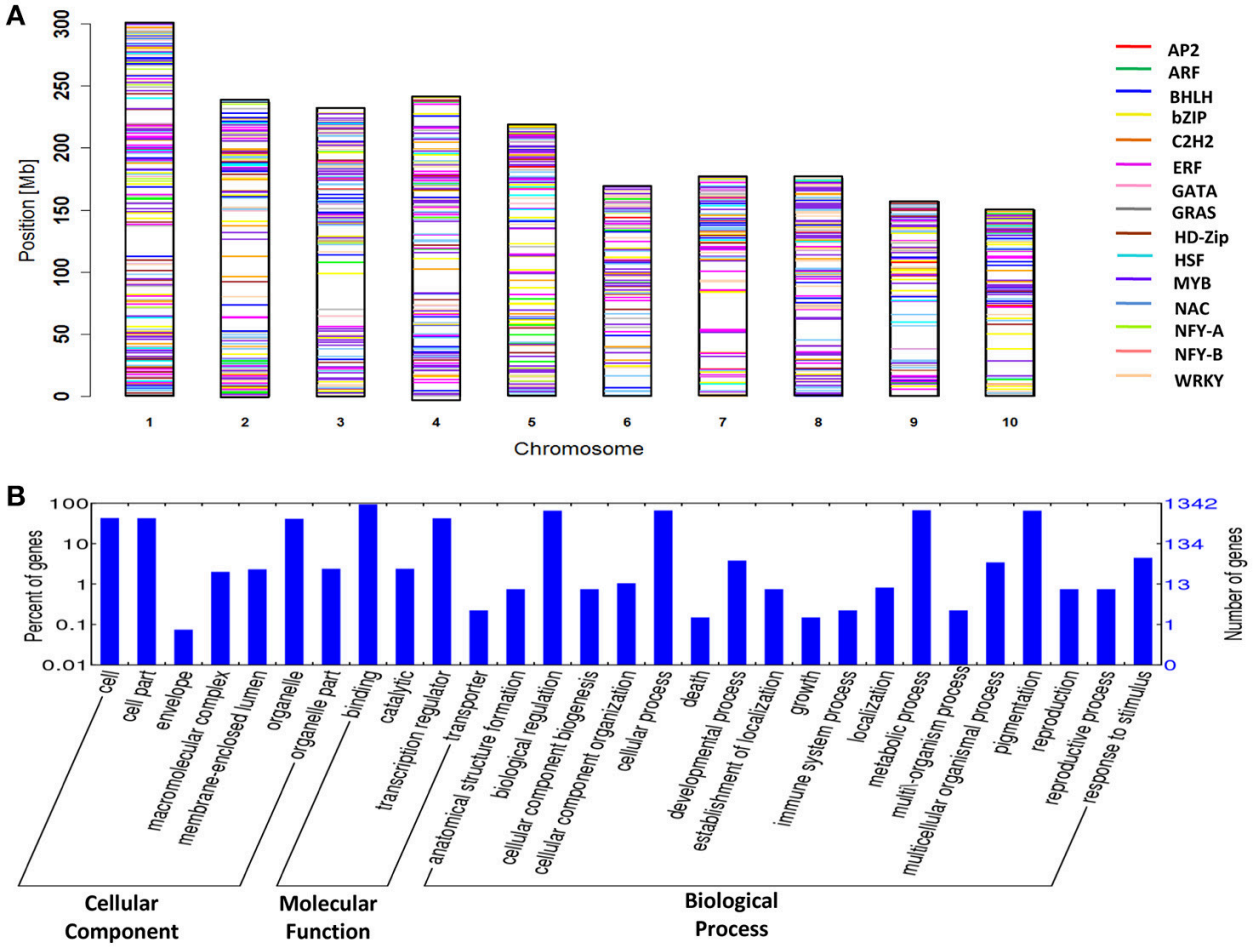
Chromosomal mapping & Blast2GO annotation of 1436 genes. **(A)** Genes associated with 15 drought-related TFs mapped on 10 chromosomes of maize according to their position. Different colors correspond to different TF families. **(B)** Genes of TF families representing various functional categories.

To investigate the genome organization of drought-related TF genes, all 1436 genes were mapped onto different chromosomes of maize. Uneven distribution of genes was found in the genome where chromosome 1 was gene-rich, containing 15% of total genes. Among all TFs, MYB family identified on chromosome 3 showed the highest gene density, whereas NF-YA showed the lowest gene density. AP2 and NF-YB is absent in chromosome 4 and 10, respectively. Chromosome 7 was devoid of the genes from ARF and GATA, and chromosomes 4, 6, 8, and 9 showed no genes from NF-YA (Figure 1A).

Genes from ARF, bHLH, bZIP, C2H2, ERF, GRAS, HD-Zip, NAC, and WRKY were discretely clustered on the chromosomes, whereas genes from AP2, GATA, HSF, NF-YA, and NF-YB, were more widely and sparsely distributed. Earlier research based on the physical and genetic framework for the reference genome (B73) has also shown such discrete distribution, which accounts for the genomic variability of the 10 chromosomes of maize and the underlying diverse changes associated with chromatin composition (Schnable et al., 2009). Additionally, movement of transposable elements during maize evolution might have contributed to the distribution of TF genes on the maize genome (Tian et al., 2009).

### Annotation of TF families

The GO annotation of TF genes was carried out to understand the involvement of the genes in different biological and molecular functions. The total GO terms identified in 1,436 genes belonging to 15 drought-related TFs were 22,506 (Supplementary Table S2). Of which, 63.30% of genes participated in biological process (BP), 40.53% in cellular components (CC), and 88.51% in molecular function (MF) (Figure 1B). Approximately 90% of the genes were predictive of their molecular function, and the most common sub-function in the molecular category was DNA binding. The GO annotations also indicated that 33% of the genes were drought-related and most of them were involved in ABA signaling, ROS scavenging, photosynthesis, stomatal development, and sucrose metabolism. The major drought-related function in ERF, WRKY, and bZIP was ABA signaling; similarly, defense and detoxification response were the major drought-related functions for MYB and C2H2. Among all genes, *DREB, ABSCISIC ACID-INSENSITIVE 5-LIKE PROTEIN, ZINC FINGER PROTEIN 2, CUP-SHAPED COTYLEDON, V-TYPE PROTON ATPase SUBUNIT H, GLUTATHIONE S-TRANSFERASE*, and *SPEECHLESS* were the important genes for stress response. It was proved that over-expression of *DREB2ca* in *Arabidopsis* and soybean improved drought tolerance without any growth impairment (Engels et al., 2013). Even in mature dry seeds, the over-expression of *ABI5* regulates *LEA* genes (Delseny et al., 2001), thus had a role in dehydration tolerance. In *Arabidopsis*, various ZFPs such as *AZF1, AZF2, AZF3, ZAT6, ZAT7*, and *ZAT10* were identified for drought-responsiveness (Sakamoto et al., 2000; Ciftci-Yilmaz and Mittler, 2008). *ZAT12* played a significant role in ROS scavenging (Davletova, 2005) thus helps in stress tolerance. *CUP-SHAPED COTYLEDON* was involved in abiotic stress tolerant mechanisms (Jogaiah et al., 2013; Wang et al., 2016). In wheat, a V-H^+^-PPase gene, *TVP1*, was identified for improving the drought tolerance (Brini et al., 2007). The role of drought-induced expression of *GLUTATHIONE S-TRANSFERASE* was well documented in rice (Sharma et al., 2014). *SPEECHLESS* and *MUTE*, belonging to the bHLH family, responded to drought stress through regulating stomatal development, meristemoid differentiation, and guard cell morphogenesis (Pillitteri et al., 2007). All drought-related genes identified by their GO annotation can be considered as candidates and could be further validated in maize genotypes.

### Phylogenetic relationships among and within drought-related TF families

**Figure 2:**
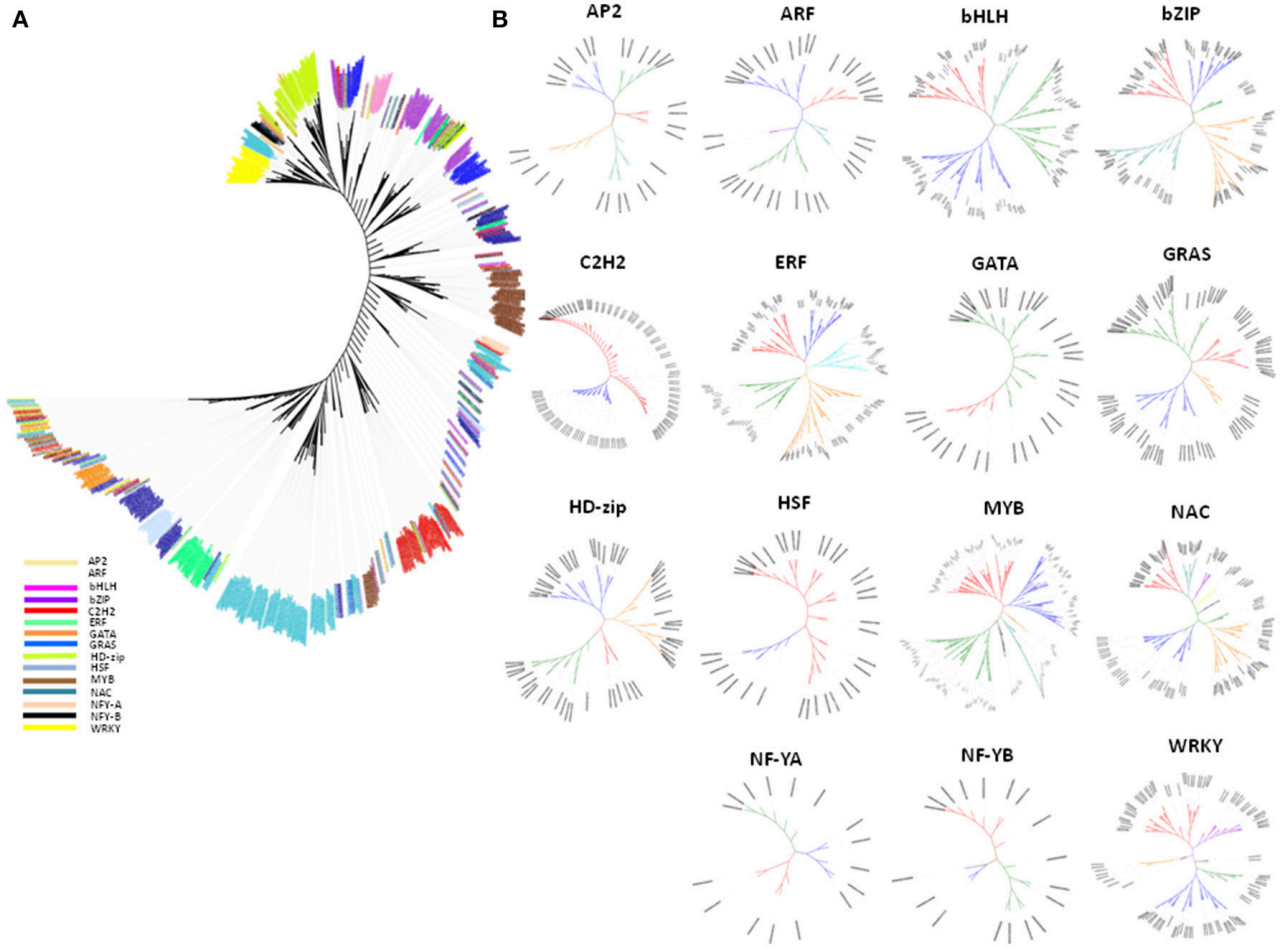
An unrooted phylogenetic tree of drought-related TF gene families. **(A)** An unrooted phylogenetic tree of the maize drought related TF families comprising of 1436 TF genes from 15 TF families. **(B)** An unrooted phylogenetic tree of TF gene families. The phylogenetic relationship of the TF genes was analyzed and depicted within the family *viz*., AP2, ARF, bHLH, bZIP, C2H2, ERF, GRAS, GATA, HD-ZIP, HSF, MYB, NAC, NF-YA, NF-YB, and WRKY.

Phylogenetic analysis was performed to understand the evolutionary relationship among and within drought-related TF families of maize. Evolutionary association among TFs indicated that the TF genes fell in the mixed clusters category, including those from the MYB family, in which about 99% were clustered into three groups. Of which two were well-differentiated (Figure 2A) while the third one was mixed with genes distributed in different clusters. The remaining 1% was present in association with their homologous genes from other TF families, including ERF, NAC, GRAS, bZIP, and C2H2. Nearly 10–15% of genes in the latter TFs were scattered outside their specific clusters, which suggested that the irregular distribution of few genes outside the TF-family could be due to their homologous association with other TF families (Figure 2A). The mixed grouping of genes belonging to various TFs suggested that these TFs could have evolved from common ancestral genes during the process of evolution. Studies have reported that mixed clustering of gene families based on sequence homology correlates with their function (Mochida et al., 2010). Several rounds of duplication at the genome level are the major contributors toward the expansion of TF families in plant evolution, and many TF families expanded in parallel with the evolution of multi-cellularity (Pires and Dolan, 2010). The lineage-specific parallel expansion of TF genes was reported in *Arabidopsis*-rice TF orthologous groups (Shiu et al., 2005). Evidences suggested that many of the eukaryotic genomes, including maize, have undergone multiple large-scale duplication events in their evolutionary history (Edger and Pires, 2009). Furthermore, joint contribution of trans-regulatory changes coupled with *cis*-regulatory changes accelerated the functional diversification of TFs (Schmitz et al., 2016). The phylogenetic association within the TF genes in maize (Figure 2B) showed that the majority of the TF trees were bifurcated into five clades (AP2, ARF, bZIP, and ERF), followed by three (bHLH, NF-YA, and NF-YB) and six clades (GRAS, HSF, and MYB). The maximum number of clades was observed in NAC TFs (10) followed by GATA and WRKY (7 each). However, C2H2 TF tree showed a significant number of polytomies and the presence of polytomies in C2H2 tree (Figure 2B: C2H2) indicated the rapid evolution of C2H2 genes. Schmitz et al. reported that repeats of sequence motifs such as C2H2 zinc fingers are responsible for the accelerated expansions of TF families (Schmitz et al., 2016).

Same TF-families does have multiple functions since they regulate different mechanisms which improve drought tolerance in maize as well as in other species. MYB family has been involved in different functions including, plant development, hormonal signal transduction, and abiotic stress tolerance (Allan et al., 2008; Cominelli and Tonelli, 2009). In maize, rice, *Arabidopsis*, and soybean, MYB members play significant role in variety of cell processes such as cell cycle and cell morphogenesis (Jin and Martin, 1999; Stracke et al., 2001; Feller et al., 2011). Different members of WRKY family play vital role in abiotic stress response, germination, plant development, and senescence (Rushton et al., 2010).

According to our results, we found that the most of the drought-related genes identified using gene expression assay were found in specific sub-clades. For example—genes belonging to HD-Zip, HSF, ERF, WRKY, and MYB were present in the cluster in different specific sub-clades of the evolutionary tree. Most of the TF genes were present in specific clades while very few genes were scattered across clades. The result indicated that the genes belonging to a specific TF group play specific function under given condition. On the other hand, the scattered ones could have shared the common functional relationship along with their respective TF group.

### Gene duplication and paralog identification

Gene duplication is considered as one of the crucial events in evolution. To elucidate the expanded mechanism of drought-related TF genes in maize during evolution process, the duplication events including tandem and segmental were studied. Duplication events are the main factor responsible for expansion of gene families. Duplication events, such as tandem and block duplications, have paved the way for new gene functions and, in turn, evolutionary freshness. The maize genome has already undergone many rounds of genome duplications, including a paleopolyploid event ~70 million years ago (Blanc and Wolfe, 2014) and a subsequent whole genome duplication event ~11 million years ago (Blanc and Wolfe, 2014). Further studies based on gene duplications in maize have confirmed that a second large duplication event is responsible for gene duplication in most of the gene families (Schnable et al., 2009). For ease of analysis, we classified all the duplication events into three types: Type I, which corresponds only with tandem duplications; Type II, only with block duplications; and Type III, with both tandem and block duplications. In the present study, the largest number of Type I, Type II, and Type III events were present in NAC (31%), HD-Zip (64%), and WRKY (10%), respectively. Type II events were more frequent than Type I events, and only 11 TFs fell into the Type III category. The range of Type I, II, and III events was 0–16.27%, 6.54–16.57%, and 6.10–15.85%, respectively. Furthermore, Type I and Type II events were prominent on chromosome 1, whereas chromosome 8 was rich in Type III events (Supplementary Figure S1). These events signify that tandem duplication was a common one for rapidly evolving families; block duplication was a feature of the slowly evolving gene families, and both played a significant role in the expansion of gene families (Liu et al., 2011).

Subsequent identification and analysis of paralogs for each TF confirmed that only 981 (68.31%) genes had paralogs out of 1,436 genes. The distribution of paralogs across all chromosomes varied from 6.62 to 16.30%: the highest number of paralogs (160 genes) was found on chromosome 1 and the lowest on chromosome 10 (65 genes).

### Gene structure and intron distribution

**Figure 3:**
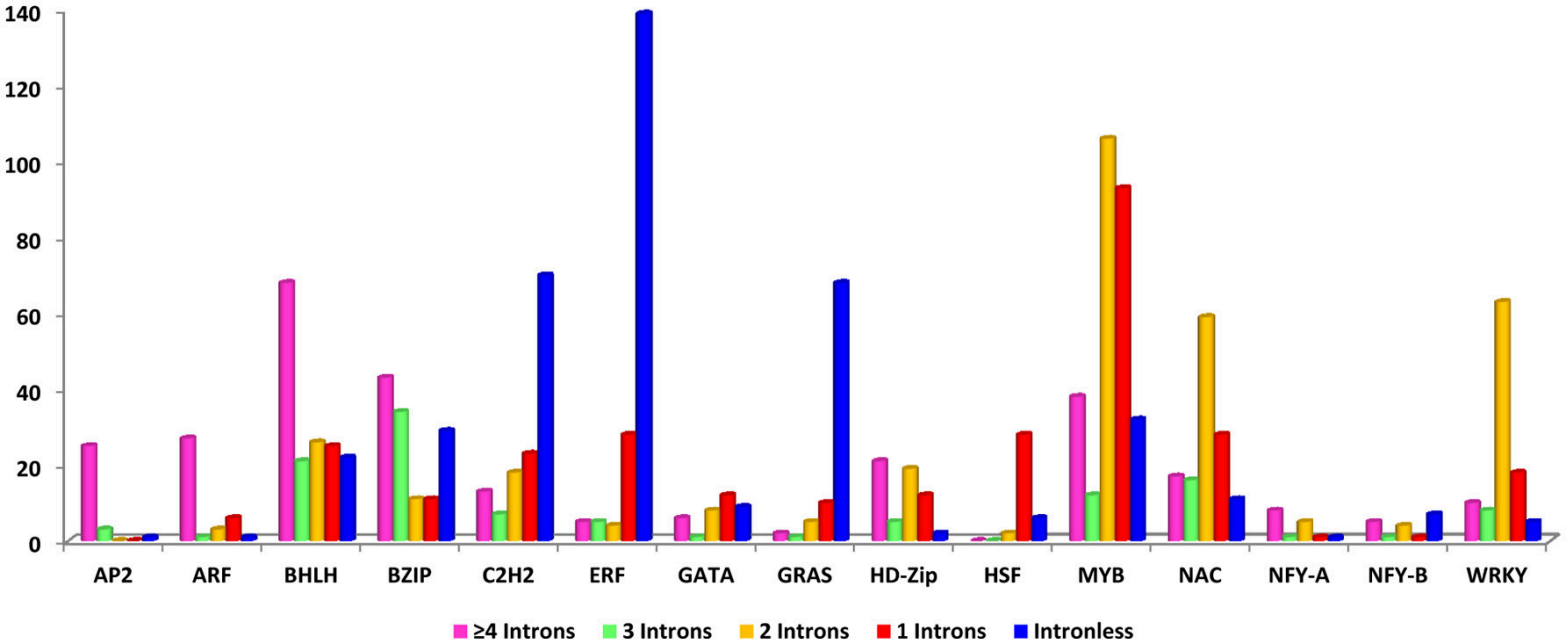
Details of gene structure: Colored bars correspond to the number of introns in each gene family; different colors are assigned on the basis of the number of introns within each gene family.

To reveal the structural features of drought-related TFs, gene structure was analyzed using GSDS server. The study of gene structure is crucial in biology and would provide important clues on gene evolution. The structural orientation of intron-exon helps to determine the evolutionary path of a gene family and provides an opportunity to investigate the evolutionary mechanisms of the origin of gene families. In multigene families, variation in gene structure forms the basis of evolution (Cao and Shi, 2012). To provide insight into this process, we determined the number of exons and introns with respect to each gene in each TF family. Further, genes were divided into intron-rich and intronless. It was found that 72% were intron-rich and 28% were intronless. The ERF family contributed to the major share of intronless genes (77%), whereas NF-YA showed only 6.25% of genes as intronless. Among intron-rich genes, 21% had a single intron, 23% had two, 8% had three, and 20% had four or more (Figure 3).

The intronless genes in eukaryotes could have evolved either through horizontal gene transfer from ancient prokaryotes or duplication of existing intronless genes and retrotransposition of intron-containing genes (He et al., 2011). The results indicated that the gain or loss of exons during evolution could be responsible for the functional diversity of these different gene families including drought tolerance. These loss and gain of introns are essential for structural diversity and complexity of genes (Zhang and Kishino, 2004).

### Conserved motifs

**Figure 4:**
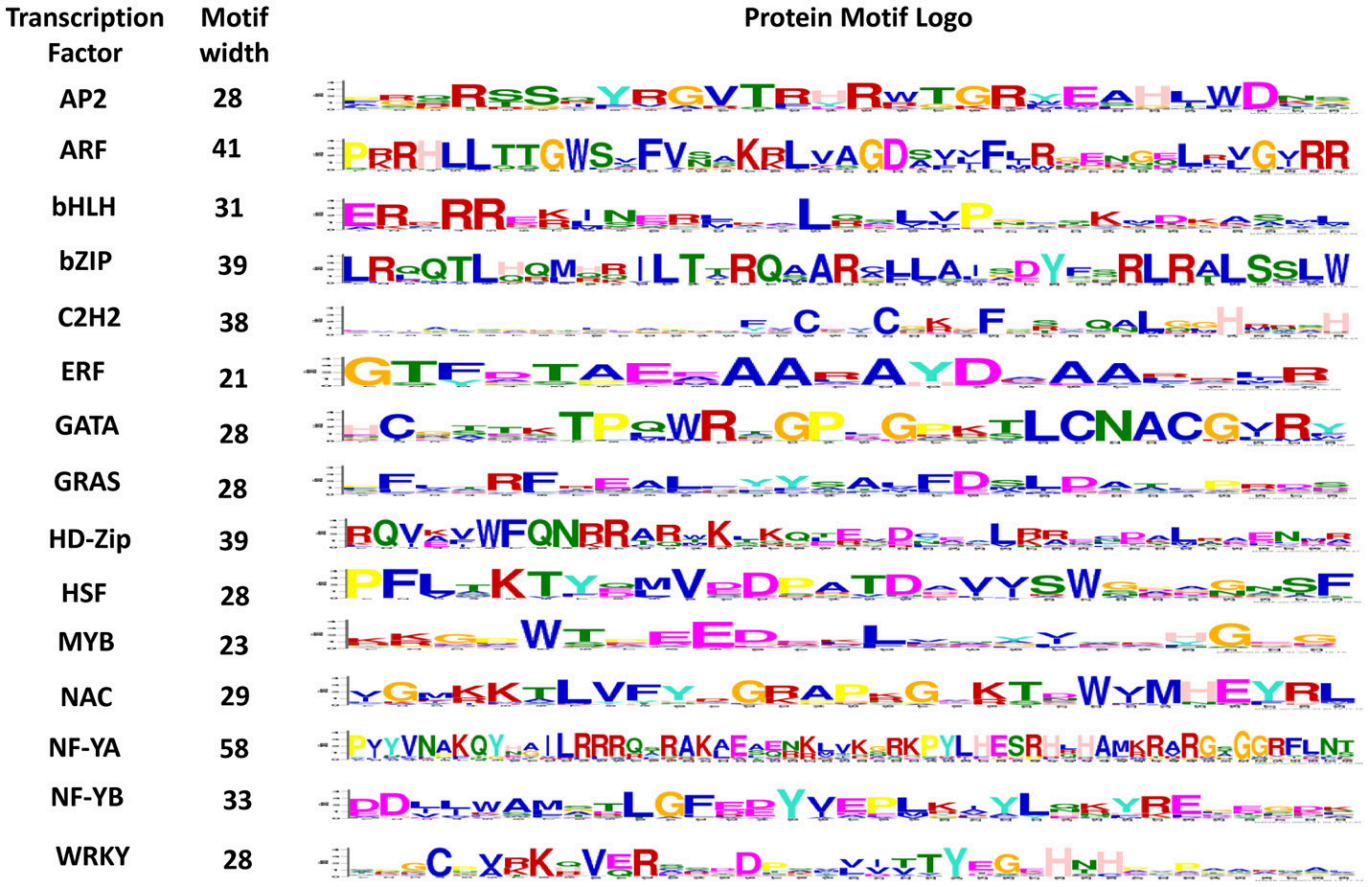
Conserved motif sequences in each of the 15 gene families of maize. The protein sequence logo for highly conserved motifs in each transcription factor is shown above.

**Figure 5:**
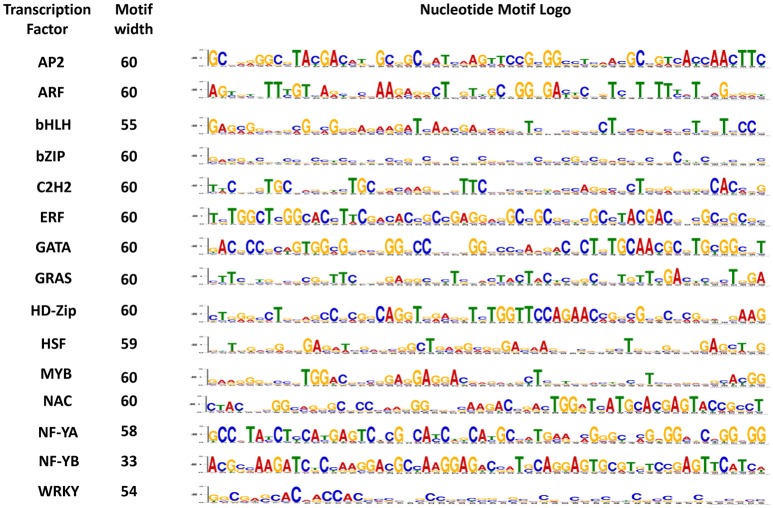
Conserved motif sequences in each of the 15 gene families of maize. The nucleotide sequence logo for highly conserved motifs in each transcription factor is shown above.

Motifs play an important role in the transcriptional regulation. To understand the similarity and diversity of gene motifs in different TF genes, five conserved motifs were identified. The importance of motifs generally lies in their association with the structure and function of proteins because protein networks are regulated by specific peptide motifs. Domain reorganization and sequence differentiation in proteins are the essential prerequisites for novel protein functions (Björklund et al., 2005). In the present study, most of the identified peptide motifs were extremely TF-specific, such as a 28-amino-acid-long sequence that was 100% conserved in all ARF genes and a 41-amino-acid-long sequence with 100% conservation in AP2 genes (Supplementary Table S3). In contrast, a 39-amino acid-long sequence conserved only in 81% genes was the least conserved sequence in HSF (Figure 4). Similarly, consistent results were obtained in nucleotide motifs: dissimilar types of 60-nucleotide-long motifs were 100% conserved in ARF, HD-Zip, bHLH, GATA, and NF-YB whereas a 28-nucleotide-long sequence conserved in 94% genes, was the least conserved motif in NF-YA (Figure 5). To gain deeper insight into the structures of conserved motifs, their composition matches were analyzed. The analysis revealed the significant features of unique and highly conserved motifs to each particular TF: for example, arginine was found at high frequencies in motif-2 (AP2), motif-2 (ARF), motif-1 (bZIP), motif 1 (HD-Zip), and motif-1 (NF-YA). In nucleotide motifs, guanine was the most conserved nucleotide across all conserved nucleotide motifs, such as motif-1 (bHLH, C2H2, GATA, GRAS, HD-Zip, NF-YA, NF-YB), motif-3 (ARF, MYB), and motif-5 (HSF). Similarly, cytosine was the second most conserved sequence in motif-1 (AP2, ERF, and NF-YB), motif-2 (NAC), motif-4 (bZIP), and motif-5 (WRKY). The conservation and differences in motif sequences between proteins represent functional similarity and diversity in terms of various aspects of biological functions (Puranik et al., 2012). Conserved motifs can provide evidence for further classification into different subgroups as it is possible that proteins within a subgroup, which share identical motifs, are likely to exhibit similar functions. Motifs information can be used to develop resistance genes and markers, perform clustering (Broin et al., 2015), assess the gene expression (Jensen et al., 2005; Huber and Bulyk, 2006), discover the homology relations, classify the families (Blekas et al., 2005; Jensen et al., 2005), discover the sub-families in large protein families (Leonardi and Galves, 2005) and identify new signaling pathways (Ma et al., 2013).

### *cis*-regulatory elements

The CREs in promoter region are the short motifs on which the TF binds to regulate the gene expression. To understand the gene regulation, the knowledge of *cis*-regulatory elements is indispensable (Mochida et al., 2011). To further investigate the transcriptional regulation and the potential functions of these drought-related TFs in maize, *cis*-regulatory motifs, a 1 kb upstream promoter region was selected in all TFs using Plant Promoter Analysis Navigator (PlantPAN). A total of 42 types of CREs were identified, of which 28 were common to all TFs (Table 1). These CREs help in determining transcriptional activity by specifying its time and location. The most abundant CRE was DOFCOREZM, with 1,168 duplications, followed by POLASIG3 andDRE1COREZMRAB17, with 1,106 and 1,059 duplications, respectively, whereas ANAEROBICCISZMGAPC4 was the least abundant, with only 134 duplications (Supplementary Table S4). The functions of CREs include responding to dehydration, sugar signaling, responding to light, fermentation and response to phyto-hormones. Of 28 CREs, only 4 were specific to drought stress. ABREAZMRAB28 (ABRE A), found at −148 to −139, and ABREBZMRAB28 (ABRE B) found at −105 to −96, were involved in ABA-responsive function in maize embryos *via* the *rab28* gene (Busk and Pagès, 1997). Other drought-related CREs, such as DRE1COREZMRAB17 (DRE1), *DRE/CRT* (dehydration responsive element/C-repeat), and DRECRTCOREAT were also found (Busk et al., 1997; Dubouzet et al., 2003). The identification of these CREs could help in understanding the diversity, distribution and evolutionary relationships of drought-related genes in maize (Wittkopp and Kalay, 2011; Hernandez-Garcia and Finer, 2014; Lemmon et al., 2014).

**Table 1:** Typical *cis*-regulatory elements found in maize with their consensus sequences and functions.

***cis*-element**	**Consensus sequence**	**Function**
−300MOTIFZMZEIN	RTGAGTCAT	Motif in−300 elements of alpha-zein genes of maize
−314MOTIFZMSBE1	ACATAAAATAAAAAAAGGCA	Sugar responsive expression in *Zea mays*
ABREAZMRAB28	GCCACGTGGG	ABA-responsive element
ABREBZMRAB28	TCCACGTCTC	ABA responsive element
ANAERO1CONSENSUS	AAACAAA	Response to anaerobic conditions
ANAERO3CONSENSUS	TCATCAC	Response to anaerobic conditions
ANAEROBICCISZMGAPC4	CGAAACCAGCAACGGTCCAG	Response to anaerobic conditions
ARECOREZMGAPC4	AGCAACGGTC	Essential for Anaerobic induction
C1MOTIFZMBZ2	TAACTSAGTTA	MYB-class TF binding site
DOFCOREZM	AAAG	Core site required for binding of Dof proteins in maize (Z.m.)
DRE1COREZMRAB17	ACCGAGA	Expression during late embryogenesis
DRECRTCOREAT	RCCGAC	Core motif of DRE/CRT (dehydration-responsive element/C-repeat)
GCAACREPEATZMZEIN	GCAACGCAAC	Recognition site for the maize endosperm-specific beta-1 factor within the promoter of the beta-zein gene
GCBP2ZMGAPC4	GTGGGCCCG	Binding site of Tobacco Nucleat Factor
IDRSZMFER1	CACGAGSCCKCCAC	Iron-Dependent Regulatory sequence
INTRONLOWER	TGCAGG	Consensus sequence for plant introns
INTRONUPPER	MAGGTAAGT	Consensus sequence for plant introns
MNF1ZMPPC1	GTGCCCTT	Involved in light induction
MYBPLANT	MACCWAMC	Plant MYB binding site
MYBPZM	CCWACC	Core of consensus maize P (myb homolog) binding site
OCSENHANMOTIFAT	ACGTAAGCGCTTACGT	OCS enhancer element
OCTAMOTIF2	CGCGGCAT	Octamer motif found in histone-gene-specific consensus sequences
OPAQUE2ZMB32	GATGAYRTGG	opaque-2 binding site of maize
POLASIG3	AATAAT	“Plant polyA signal”; Consensus sequence for plant polyadenylation signal
QELEMENTZMZM13	AGGTCA	Involved in expression enhancing activity
RYREPEAT4	TCCATGCATGCAC	Involved in seed expression and ABA
SPHZMC1	CGTCCATGCAT	Essential for VP1 activation
TATAPVTRNALEU	TTTATATA	Binding site of TATA binding protein (TBP)

### Interaction among *cis*-regulatory elements and TF assigning drought tolerance in plants

**Figure 6:**
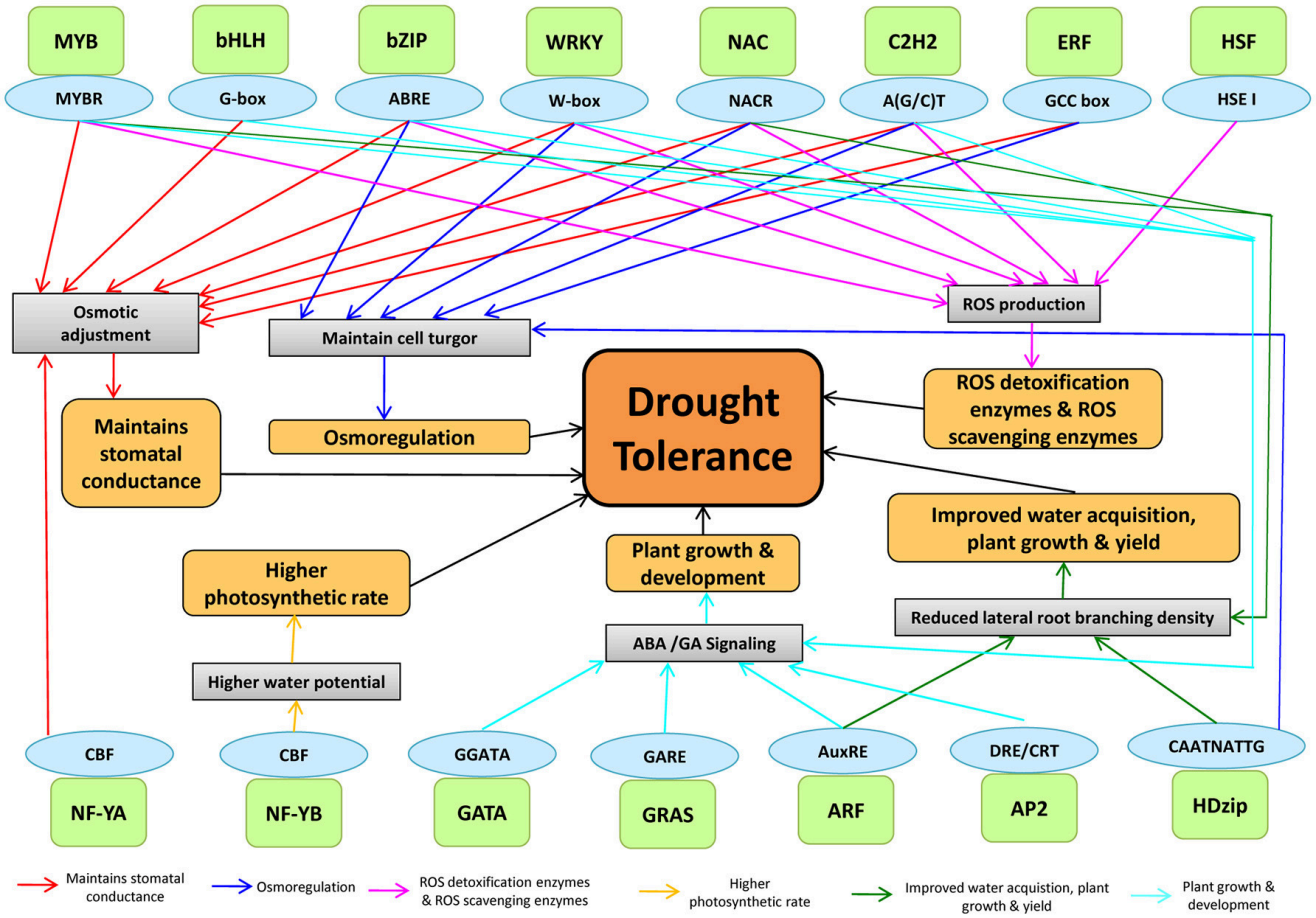
The genome-TF interaction in assigning drought tolerance in plants. The TFs binds to *cis*-acting elements to modulate the physiology of plants under drought stress. A light green color box indicates TFs, and the sky-blue color indicates their *cis*-acting elements.

Plants respond to drought stress at physiological and molecular levels through complex processes, mediated by TF interactions (Figure 6). The drought-tolerant mechanisms are regulated through TFs by binding to different *cis*-acting elements in maize (Figure 6). DREB, AREB/ABF, MYB, WRKY, and ERF bind to *cis*-regulatory elements such as DRE/CRT, ABRE, MYBRS, W-boxes, and GCC-boxes, respectively that are present in the promoter region of drought-responsive genes to regulate the gene expression (Nakashima et al., 2007). For example- MYB by binding to MYBR regulates different drought tolerant mechanisms including stomatal closure, ROS detoxification, and scavenging. ARF by binding to AuxRE regulates plant growth and development and reduced lateral branching. NAC was involved in maintaining cell turgor pressure, ROS homeostasis and plant development. NF-YB was involved in controlling higher photosynthetic rate by binding with CBF *cis*-element. While AP2, ARF, GRAS, GATA, C2H2, WRKY, MYB, bHLH, and bZIP were found to regulate plant growth and development *via* ABA/GA signaling.

The interaction of TFs in ABA-dependent and ABA-independent pathways are known to regulate the plant transcriptional response under drought stress by activating stress-responsive genes, resulting in stomatal closure to check excessive water loss by transpiration (Nakashima et al., 2014). The involvement of *AtMYB60* and *AtMYB61* in stomatal regulation was clearly demonstrated under drought stress (Cominelli et al., 2005; Liang et al., 2005). In rice, *SNAC1* and *DST* (a C_2_H_2_-type Zinc finger-containing protein) were involved in stomatal opening/closure (Hu et al., 2006; Huang et al., 2009). The interaction between AREB/ABF and NAC was previously reported under drought stress. *ATAF1*, a *SNAC* TF, binds to the promoter of *NCED3*, which directly controls ABA hormone levels, suggested the role of *SNAC* in regulating ABA-dependent gene expression (Jensen et al., 2013). In response to drought and osmotic stress, it was reported that *ANAC096* cooperated with *ABF2* and *ABF4* factors (Xu et al., 2013).

Drought stress increases the water-use-efficiency (WUE) through the promotion of deeper root system and inhibition of lateral root development to maximize the water absorption from deeper soil layers (Overvoorde et al., 2010). During drought, several NAC genes regulate the root morphology to enhance the WUE in plants (Jeong et al., 2010). Detoxification of ROS formed during drought stress is important to adjust the physiology of the plants to cope up with the stress. ROS accumulation is dependent on the equilibrium between ROS scavenging and ROS production (Mittler et al., 2004). Antioxidants, such as ascorbic acid, glutathione, and ROS-scavenging enzymes such as glutathione peroxidase, superoxide dismutase, and ascorbate peroxidase, are required for ROS detoxification (Mittler et al., 2004). In rice, several genes, including NAC (Hu et al., 2006), DST (Huang et al., 2009), MYB (Dai et al., 2007), and ZIP (Xiang et al., 2008), play significant roles in drought tolerance through ROS scavenging. C2H2-type zinc finger proteins play vital role in the synthesis of osmoprotectants and detoxification of ROS (Davletova, 2005). WRKY genes were suggested to be involved in ROS detoxification and ROS scavenging. Crosstalk between ROS signaling, ABA-signaling, and other stress-induced redox and metabolite signaling has also been observed. In wheat, to prevent oxidative damage in plant cells, *WRKY44* decreases ROS levels (Wang et al., 2015). Cross-talks between AREB with DREB led to the complex interaction of TF families (Narusaka et al., 2003; Lee et al., 2010; Kim et al., 2011). In *Arabidopsis, ANAC096* interacts with *AREB* to provide the drought tolerance (Xu et al., 2013) and interaction between *DREB* and AP2/ERF has also been reported (Cheng et al., 2013). *OsMyb4* integrated with ABA and bZIP that directly regulates the ABA-responsive genes (Nakashima et al., 2009). It was also reported that NF-YB and NF-YC form a dimer and interacts with NF-YA in the nucleus and plays prominent role in root development (He et al., 2016). Understanding the inter-connection among the drought tolerant mechanisms through TFs regulation would help to breed drought-tolerant maize in the changing climatic scenario in a more efficient way. The information could be also useful in short-listing the best combination of genes that work in tandem under drought stress condition for further exploitation.

### Expression analysis of drought-responsive TFs genes under drought

**Figure 7:**
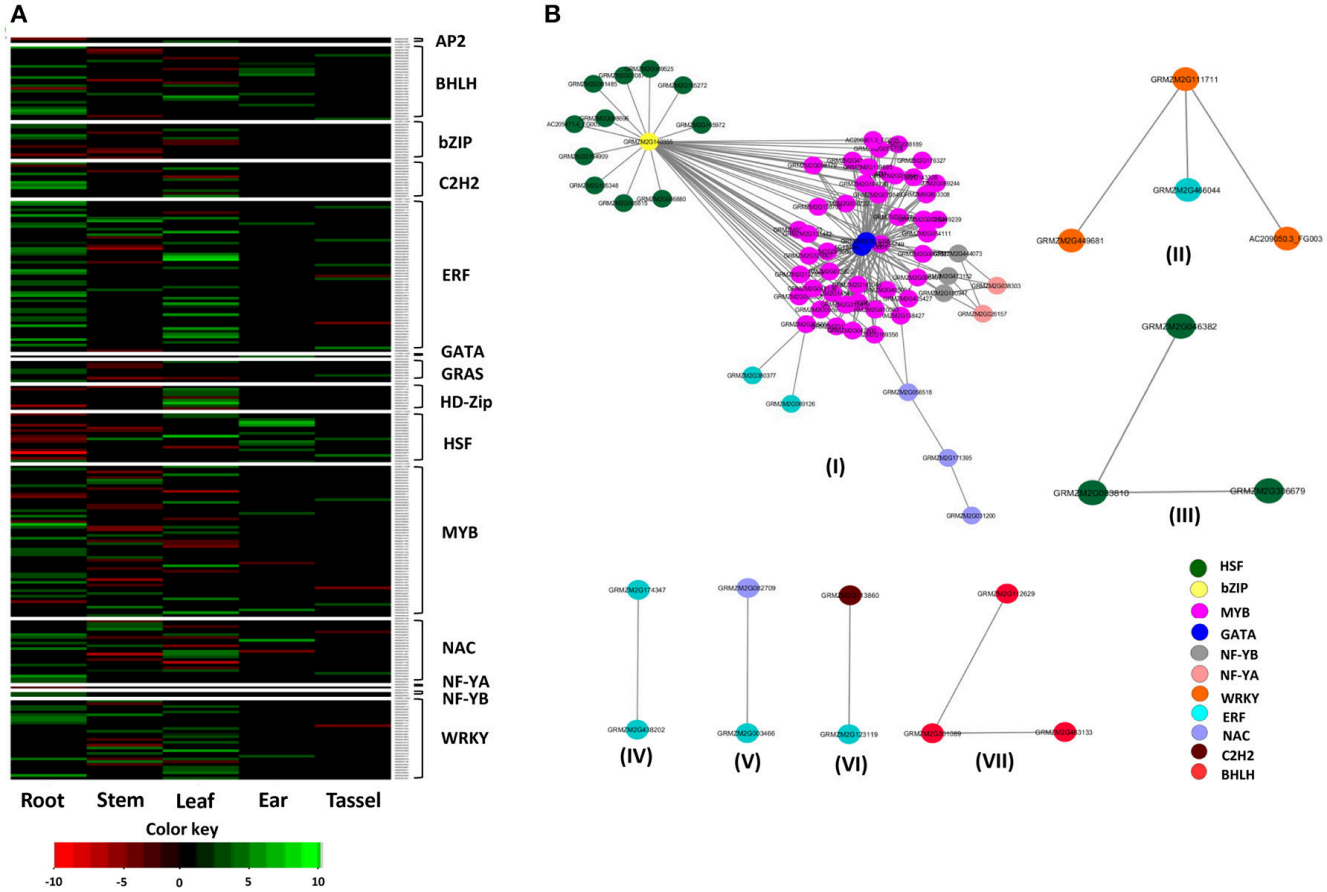
Heatmap and interaction network of 269 drought-responsive genes. **(A)** Overview of differentially expressed TF genes in response to drought stress in various tissues with log2-fold changes of 2 or more and with a *p-*value of <0.05. A total of 269 DEGs from 14 of 15 TF families were represented in different tissues. **(B)** Drought-responsive TF network of 269 genes. A total of seven networks were identified. The color of the nodes indicates the category of TFs.

To understand the expression pattern of TF families in response to drought stress, RNA-Seq data available in the public domain were used. Several reports have discussed the role of drought-responsive TFs genes in the modulation of drought tolerance through differential expression patterns in tissue-specific and generic manners (Gahlaut et al., 2016). To investigate the expression pattern of these TFs families under drought stress, the TF genes were considered from the experiments of Liu et al. (2015) and Thatcher et al. (2016). A total of 269 genes belonging to 14 TF families with fold change of >2 was selected for further analysis (Figure 7A). Results explained that the genes of the ARF family did not show any differential expression under drought stress conditions. The maximum number of differentially expressed genes was in ERF and MYB families (56 each) and the least was in AP2, GATA, and NF-YA (2 each). Furthermore, among tissues, roots showed maximum number of differentially expressed genes (DEGs) (109), followed by leaves (92), shoot (72), ear (22) and the lowest was in the tassel (18). No common TF genes were differentially expressed in the tissues, indicating the tissue specificity in TF gene expression. Genes were over-expressed in response to drought stress in the majority of the TFs families. It was noted that the roots regulate drought stress by plant hormones and ABA pathways. An early response to drought stress induces ABA in roots, which further transmits signals from roots to aerial parts through a cascade of reactions (Janiak et al., 2016). In roots, drought stress induces up-regulation of TF genes was reported in several plant species, including rice, maize, and wheat (Janiak et al., 2016; Opitz et al., 2016; Zhang et al., 2016). Shiriga et al. (2014) reported an up-regulation of NAC genes (*GRMZM2G336533* and *GRMZM2G347043*) in the tolerant genotype (HKI577) under drought stress compared to the sensitive genotype (HKIPC3-3). It was demonstrated that the down-regulation of *ATHB7*, a member of HD-Zip, was associated with drought-induced ABA signal transduction (Gao et al., 2015). Furthermore, several TF genes did not show any differential expression pattern in the selected tissues due to an environmental condition, genetic background effect and/or growth stages of the plant (Wang et al., 2017). Among bZIP genes, *GRMZM2G140355* showed leaf-specific down-regulation under drought stress which was also confirmed by Jiang et al. (2012).

The role of TF families under drought stress has been documented in several crops. For example- the over-expression of *DREB1/CBF* improved drought tolerance in tomato, chrysanthemum and potato (Iwaki et al., 2013), soybean (de Paiva Rolla et al., 2014), rice (Nakashima et al., 2014; Paul et al., 2015), tobacco (Phuong et al., 2015), sugarcane (Augustine et al., 2014), groundnut (Tiwari et al., 2015), peanut (Bhatnagar-Mathur et al., 2014), and wheat (Shavrukov et al., 2016). Majority of the *DREB* genes were involved in ABA-independent stress responses whereas ABA-dependent responses was also reported (Sakuma et al., 2006; Nakashima et al., 2009). In rice, regulation of downstream gene *OsPP18* by *SNAC1* conferred drought tolerance *via* ABA-independent pathway (You et al., 2014). Over-expression of *ANACO19, ANACO55*, and *ANACO72* also explained drought tolerance in *Arabidopsis*. In barley, *HvSNAC1* not only imparted drought tolerance but also provided resistance against leaf spot (Al Abdallat et al., 2014; McGrann et al., 2015). *AtMYB60* and *AtMYB96* were involved in drought response and regulate stomata and disease resistance *via* ABA-dependent pathways (Cominelli et al., 2005; Seo et al., 2009). In rice, *OsWRKY45* was up-regulated under drought stress treatment and involved in ABA synthesis that induces a signaling cascade resulting in enhanced drought tolerance (Qiu and Yu, 2009; Supplementary Table S5).

### Interaction of drought-responsive TFs in assigning drought tolerance

Protein-protein interaction is an important component of gene regulation and is known to mediate most of the cellular processes (Ding et al., 2014). The interactions among different TFs are the major sources of diversity in the gene expression pattern. The physical interactions among multiple TFs may mediate the specific set of conditions under which gene expression will be regulated (Reményi et al., 2004; Hussain et al., 2011). Therefore, understanding the interactions of various drought-responsive TF proteins provides a deeper understanding of molecular mechanisms and pathways operating in drought tolerance. A set of 269 genes showing variable expression levels under drought stress in different tissues of maize was selected for interaction analysis. The results revealed the presence of seven networks with 269 nodes and 212 edges with a clustering coefficient of 0.79 (Figure 7B). Network I showed maximum number of interfamily interactions among the TF families, including bZIP, GATA, HSF, MYB, NF-YB, and NF-YA.

In Network I, bZIP (*GRMZM2G140355*) showed maximum interactions (54), including 11 HSF and 43 MYB. From the phylogenetic tree (Figure 2), the MYB genes are grouped together with bZIP genes. Similarly, the HSF genes were grouped in a single cluster. The grouping of genes of TF families suggested that they could be involved in similar functions under drought stress. MYB, HSF, and bZIP explained the higher level of structural relationship since more than 95% of the *cis*-elements were same. From an evolutionary perspective, among different TF families, it has been noticed that some TF families have higher rate of expansion including bZIP, BHLH and MYB over others (Shiu et al., 2005; Feller et al., 2006; Riechmann et al., 2016). The interactions among the bZIP and MYB in *Arabidopsis* are known to provide light responsiveness in plants and induce stress signaling pathways (Hartmann et al., 2005). bZIP and MYB, along with tobacco bZIP transcription activator (TAF), play direct roles in drought tolerance through ABA signaling pathway (Golldack et al., 2011). *OsMyb4* was integrated with ABA and other signaling pathways through bZIP that directly regulates the ABA-responsive genes (Nakashima et al., 2009). The GATA genes, *AC194965.4_FG004*, and *GRMZM2G113098* interacted with MYB and NF-YB families (IN WHICH CROP OR WHAT PURPOSE/ROLE). During seed germination, a repressor of *gal-3* (*RGA*) and *RGA-LIKE2* (*RGL2*) interacts with NF-YB proteins to regulate GA, ABA, and GA signaling pathways (Hou et al., 2014; Liu et al., 2016). NF-YA (*GRMZM2G026157, GRMZM2G038303*) interacted with six NF-YB proteins (*GRMZM2G180947, GRMZM2G444073, GRMZM2G473152, GRMZM2G180947, GRMZM2G444073*, and *GRMZM2G473152*) (IN WHICH CROP OR WHAT PURPOSE/ROLE). The interaction of NF-YA and NF-YB families could be attributed to the high level of similarity of *cis*-elements (98%) between them. Several NF-YA and NF-YB have been found to play significant role in drought tolerance (Nelson et al., 2007; Li et al., 2008; Hackenberg et al., 2012). The NF-YA (*GRMZM2G026157*) and its interaction with other co-factors play a prominent role in the development of roots under stress (He et al., 2016). It has also been reported that NF-YB and NF-YC form a dimer and further by interacting with NF-YA, a trimer is formed in the nucleus. In the promoter of its target genes, this trimer binds to *cis*-elements called CCAAT-box and further regulates the downstream stress-related genes (Frontini et al., 2004; Kahle et al., 2005). It has been reported that *TaNF-YA10–1* by interacting with NF-YA/NF-YB activates target drought-responsive genes (Ma et al., 2015).

### Utility of drought-related TFs in stress breeding

TFs are the potential regulators of various genes that help plants to effectively cope up with drought stress. To unravel the molecular mechanisms in response to drought stress, it is important to mine candidate genes, pathways, and the interaction of genes in different pathways. Genetic manipulation of combination of drought stress-responsive TF genes stands to be a powerful approach for improving stress tolerance than addressing each functional gene individually. DREB, MYB, and bZIP are the important TFs which have been already used in several molecular breeding programmes (Foolad et al., 2003; Fleury et al., 2010). Genes involved in ABA biosynthesis, catabolism, and signaling network represent interesting candidate genes for breeding crops with improved drought tolerance. Different candidate genes that are involved in the detoxification process have been reported to benefit plant growth as well as survival upon abiotic stress and therefore provide an excellent resource for improving drought tolerance in crop breeding.

The major emphasize of the experiment is laid on detailed characterization of drought-related TFs and their structural and functional importance in assigning the drought tolerance. It should be noted that only a low level of phenotypic variation (for example 10–30%) could be achieved through transfer of a major gene or QTL since drought tolerance is a complex trait controlled by many genes (Thirunavukkarasu et al., 2014, 2017). On the other hand, by selecting a combination of genes that are involved in related pathways would provide enhanced stress tolerance. Our experiment provided comprehensive information of TF genes that are related to stress tolerance and their interactions. Presently, the breeding strategies for quantitative traits such as drought are re-oriented from a few genes (through marker-based selection) to genome-wide approaches (through genomic selection) to capture the total phenotypic variation. The identified set of TFs could be targeted as candidates to breed drought tolerant maize genotypes through different selection approaches.

## Conclusion

TFs play a key role in the regulation of drought tolerance in crop plants. An effort has been made to characterize the major drought-related TF families encoded by 1,436 genes in maize. The emphasis of the study was to understand the structure, function, and evolution of the drought-related TF families and their utility in maize drought breeding. An extensive *in-silico* analysis was performed to understand the distribution, structure, expression pattern, interactions, and function in maize. Our results linked the structural characteristics of different TFs with the functional aspects in drought-stress tolerance. The working hypothesis revealed the interaction of TFs with various molecular functions was crucial in assigning drought tolerance in maize. The identified set of TFs could be targeted to breed drought-tolerant maize genotypes through selection approaches, such as genomic selection, to achieve maximum phenotypic expression. The present study thus attempts to provide a novel foundation for such comparative analysis of TF families and the findings would accelerate the understanding of drought tolerance mechanisms in maize in addition to their practical utility in drought breeding programs.

## Author contributions

NT and SM conceived and designed the experiments. SM, PB, MM, AR, and PJ analyzed the data. NT, SM, MM, PB, and PD drafted the manuscript. All authors read and approved the final manuscript.

### Conflict of interest statement

The authors declare that the research was conducted in the absence of any commercial or financial relationships that could be construed as a potential conflict of interest. The reviewer, SK, and handling Editor declared their shared affiliation.
