# Supplementary material for: Structural, Functional, and Evolutionary Characterization of Major Drought Transcription Factors Families in Maize
Source: Front Chem. 2018 May 23;6:177. doi: 10.3389/fchem.2018.00177 (PMC5974147; doi:10.3389/fchem.2018.00177)
Supplement: Supplementary file 6 [file Image_1.PDF]

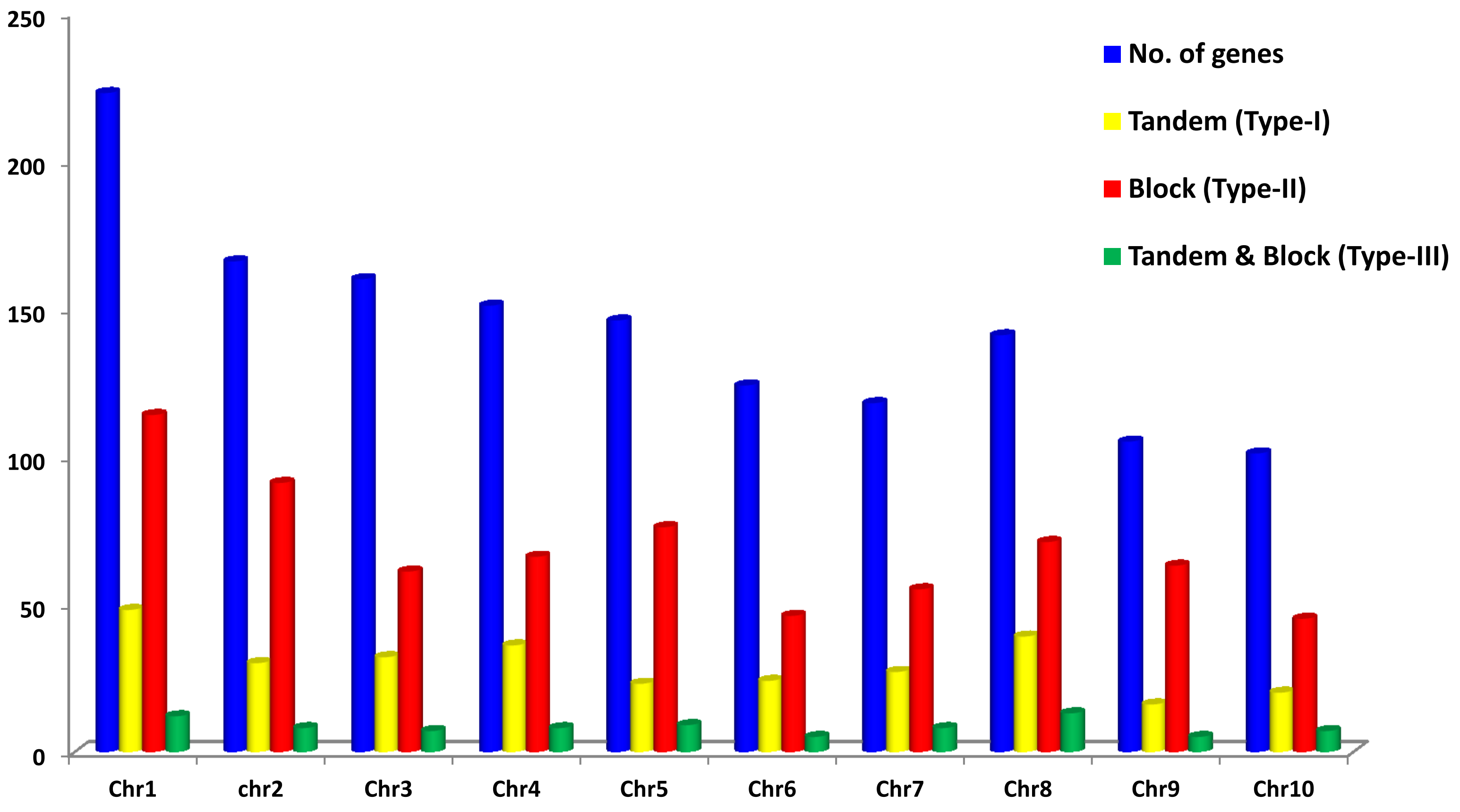

Supplementary Figure S1: Duplication events: Tandem and block duplication events on 10 chromosomes of maize against the total number of genes on each chromosome.
